# Penile metastasis from primary transitional cell carcinoma of the renal pelvis: first manifestation of systemic spread

**DOI:** 10.1186/1471-2407-4-90

**Published:** 2004-12-03

**Authors:** Giorgio Pomara, Ilaria Pastina, Maurizio Simone, Paolo Casale, Gabriella Marchetti, Francesco Francesca

**Affiliations:** 1 Department of Surgery, Urology Unit, S. Chiara Hospital, Pisa, Italy; 2 Department of Oncology, S. Chiara Hospital, Pisa, Italy; 3 Department of Pathological Anatomy, S. Chiara Hospital, Pisa, Italy

## Abstract

**Background:**

Almost one-third of all penile metastases are detected at the same time as a primary tumor, whereas the remaining two-thirds are detected a mean of 18 months after the discovery of the primary tumor. Cutaneous metastasis of transitional cell carcinoma (TCC) is extremely rare and generally accepted as the late manifestation of a systemic spread.

**Case presentation:**

We report the first case of simultaneous penile and lung metastases from a primary TCC of the renal pelvis in a 76-year-old man, that occurred 8 years after a left nephroureterectomy.

**Conclusions:**

This case report underscores the importance of physical examinations of the skin of patients who undergo surgical procedures for TCC from bladder as well as from the upper urinary tract, including those seemingly without metastatic disease, because of the possibility of skin and penile metastatic spread.

## Background

Approximately 300 cases of penile metastases are reported worldwide [1]. Seventy percent of these metastases have their origin in the genitourinary tract, and the primary tumor is most frequently located in the bladder [2]. We present the first case of penile metastasis from primary transitional cell carcinoma (TCC) of the renal pelvis, and discuss the striking feature of discovering lung metastasis almost simultaneously.

## Case presentation

**Figure 1:**
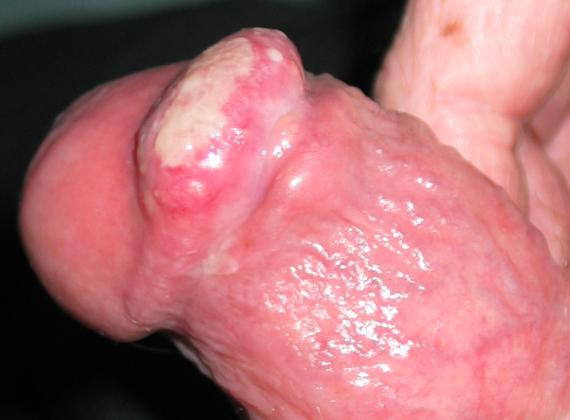
Appearance of the metastatic swelling at the corona glandis with erythema and erosion of the glans.

**Figure 2:**
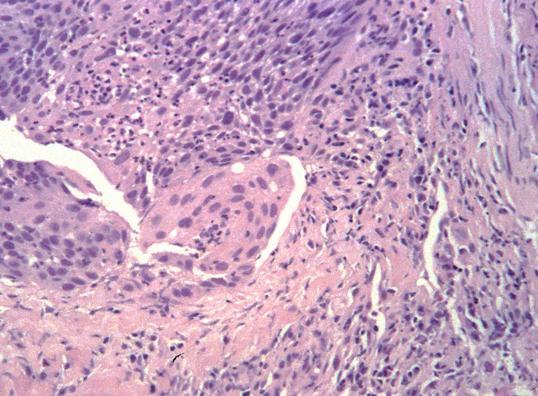
Microscopic appearance of the penile lesion demonstrating a nest of atypical malignant cells (hematoxylin-eosin stain).

A 76-year-old man with a painful, ulcerative swelling of the glans that had appeared less than 2 months previously was brought to our attention. Physical examination revealed a 3.5-cm fungating lesion involving the upper half of the glans [Fig. 1]. The lesion was malodorous with varying amounts of seropurulent discharge, partially ulcerated, erythematous, and slightly itchy. The superficial inguinal lymph nodes were not palpable. No similar lesions were evident on the head, neck, trunk, or arms. The patient had been treated in another hospital 8 years previously for TCC of the left renal pelvis by nephroureterectomy (pT2 Nx M0 G2). Subsequent postoperative follow-ups (every 6 months for the first 3 years after surgery and then once every year) consisting of a clinical examination, total body CT scan, and regular endoscopic examination showed no evidence of recurrence and the patient did well until he noticed the painful penile nodule. Pathological examination of incisional biopsies confirmed the lesion to be urothelial carcinoma [Fig. 2]. A subsequent CT workup revealed a 4-cm lesion localized in the upper-right pulmonary lobe and enlarged pelvic lymph nodes suggestive of multiple metastases.

The patient began combination chemotherapy (gemcitabine at 1250 mg/m2 on days 1, 8, and 15, and then every 28 days for six courses) and external beam radiotherapy to the mass, which promptly relieved the penile pain. At an 8-month follow-up the patient was still alive with no remarkable changes to the pulmonary and lymph node metastases, or the penile swelling.

## Conclusion

Cutaneous metastases from primary TCC of the urinary system are extremely rare and are generally accepted as late manifestations of systemic spread [1]. Spector et al. (1987) suggested that skin metastases in TCC were the result of increased longevity in successfully treated patients [3]. Various mechanisms by which lesions may metastasize to the penis have been reported. Recently Bordeau and Lynch and Berger et al. reported three cases of penile metastases from TCC of the bladder in which they assumed that metastatic spread from primary bladder cancer to the penis occurred mainly via the retrograde venous route [2,4]. There are also a few reports of TCC seeding outside the urinary tract after iatrogenic procedures (i.e., partial cystectomy, suprapubic cystostomy, pyelotomy, and laparoscopy) as the cause of cutaneous metastasis [1,5,6]. Between 5% and 20% of patients with superficial bladder cancer have vascular or lymphatic spread [5], and the reported rate of skin metastasis from TCC of the bladder is between 0.2% and 2% [7]. The rate of skin metastasis from TCC of the renal pelvis is currently unknown, and to the best of our knowledge ours is the first reported case of penile metastasis from TCC of the upper urinary tract.

Almost one-third of all penile metastases are generally detected at the same time as a primary tumor, whereas the remaining two-thirds are detected a mean of 18 months after the discovery of the primary tumor [8]. Because the disease-free survival of this patient was 8 years following the diagnosis of the primary tumor, this case report represents a description of the biology of an unusual tumor. Moreover, the penile metastasis in this case was not the late manifestation of systemic spread, as is usually reported in the literature [1]; in fact, the patient was accurately evaluated with a standard follow-up scheme. Therefore, it is plausible that the lung metastasis appears almost simultaneously with the penile metastasis.

The optimal treatment of penile metastasis requires a multidisciplinary approach that is correlated with the disease extent. The average survival in patients with penile metastasis is 3.9 months from diagnosis and, with extensive surgery and chemotherapy, a survival of 9.2 months has been reported [4].

This patient was not eligible for platinum-based regimens due to impaired renal function (prior nephrectomy) and advanced age. As a result, he was treated with gemcitabine as a single agent. This treatment has demonstrated objective response rates of 25–29% with minimal toxicity as compared to standard regimens [9-11].

In conclusion, this case report suggests that particular attention should be paid to physical examination of the skin of patients who undergo surgical procedures for TCC from bladder as well as from the upper urinary tract, including those seemingly without metastatic disease, because of the possibility of skin and penile metastatic spread.

## Competing interests

The author(s) declare that they have no competing interests.

## Authors' contributions

GP drafted the manuscript and coordinated the co-authors. IP, MS, PC participated in the sequences alignment. GM carried out the histological features of the lesion. FF participated in the sequences alignment and coordinated the co-authors. All authors read and approved the final manuscript.

## Pre-publication history

The pre-publication history for this paper can be accessed here:
